# Relationships Between Weight, Physical Activity, and Back Pain in Young Adult Women

**DOI:** 10.1097/MD.0000000000003368

**Published:** 2016-05-13

**Authors:** Sharmayne R.E. Brady, Sultana Monira Hussain, Wendy J. Brown, Stephane Heritier, Baki Billah, Yuanyuan Wang, Helena Teede, Donna M. Urquhart, Flavia M. Cicuttini

**Affiliations:** From the Department of Epidemiology and Preventive Medicine, School of Public Health and Preventive Medicine, Monash University, Melbourne, Victoria (SREB, SMH, SH, BB, YW, DMU, FMC); School of Human Movement and Nutrition Sciences, University of Queensland, St. Lucia, Queensland (WJB); Monash Center for Health Research and Implementation, School of Public Health and Preventive Medicine, Monash University (HT); and Diabetes and Vascular Medicine Unit, Monash Health, Melbourne, Victoria, Australia (HT).

## Abstract

Back pain causes enormous financial and disability burden worldwide, which could potentially be reduced by understanding its determinants to develop effective prevention strategies. Our aim was to identify whether modifiable risk factors, weight and physical activity, are predictive of back pain in young adult women.

Women born between 1973 and 1978 were randomly selected from the national health insurance scheme database to participate in The Australian Longitudinal Study of Women's Health. Self-reported data on back pain in the last 12 months, weight, height, age, education status, physical activity, and depression were collected in 2000, 2003, 2006, 2009, and 2012. In 2000, 9688 women completed the questionnaire and 83% completed follow-up 12 years later.

At baseline, median age was 24.6 years and 41% had self-reported back pain. For every 5 kg higher weight at baseline, there was a 5% (95% confidence interval [CI] 4%–6%) increased risk of back pain over the next 12 years. Higher weight at each survey also predicted back pain risk 3 years later (*P* < 0.001). The effects of weight on back pain were most significant in those with BMI ≥25 kg/m^2^ and were observed at all levels of physical activity. Inadequate physical activity and depression were independent predictors of back pain over the following 12 years (both *P* < 0.001), after adjusting for age, weight, height, and education status.

Back pain is common in community-based young adult women. Higher weight, inadequate levels of physical activity, and depression were all independent predictors of back pain over the following decade. Furthermore, the adverse effects of weight on back pain were not mitigated by physical activity. Our findings highlight the role of both higher weight and physical inactivity in back pain among young women and suggest potential opportunities for future prevention.

## INTRODUCTION

Back pain is a major global health problem, causing greater disability worldwide than any other condition.^1^ The total costs of back pain in the United States of America have been estimated to exceed $100 billion USD per year, with much of these costs being attributed to indirect costs, such as lost wages and reduced productivity.^2^ Not only is back pain associated with significant economic burden^3^ and major disability, but therapeutic options also have limited efficacy.^4^ Hence, clarifying predictors of back pain is important to optimize preventive strategies. Furthermore, as previous episodes of back pain are predictive of future recurrence,^5,6^ understanding risk factors associated with back pain in early adulthood is particularly important.

There is a lack of scientific consensus about the root causes of back pain, and there is poor correlation between back pain and spinal imaging.^7^ There are several potential risk factors for back pain that have been previously described, including female sex, white ethnicity, age, and psychological factors.^8^ Not only are women in many studies more likely to suffer from back pain,^9–11^ they also have been reported to utilize health care to a greater extent than men.^12,13^ However, few studies in younger women have addressed potentially modifiable risk factors for back pain, such as obesity and physical activity. In a prospective study of the 1958 British birth cohort, self-reported obesity in women at age 23 increased the incidence of back pain 10 years later (adjusted odds ratio [OR] 1.78).^14^ However, in a subsequent article based on this cohort, obesity was not a risk factor for incident back pain from 32 to 33 years of age.^15^ Other cohort studies of young adolescent twins^16^ and young adults without back pain^17^ have shown either no or minimal association between overweight or obesity and nonspecific back pain several years later. Similarly, in recent meta-analyses, although cross-sectional studies have reported associations between obesity and back pain, there is a lack of longitudinal data to support a temporal relationship.^18,19^ Physical activity may also have a role in back pain, but to date, prospective studies examining its relationship with back pain have yielded inconsistent results^17,20^ with none addressing this question in young community-based women in systematic reviews.^21,22^

Thus, the aim of this study was to determine whether weight and physical activity predict future back pain in young women over a 12-year period, in a large population-based cohort study.

## METHODS

### Participants

**FIGURE 1:**
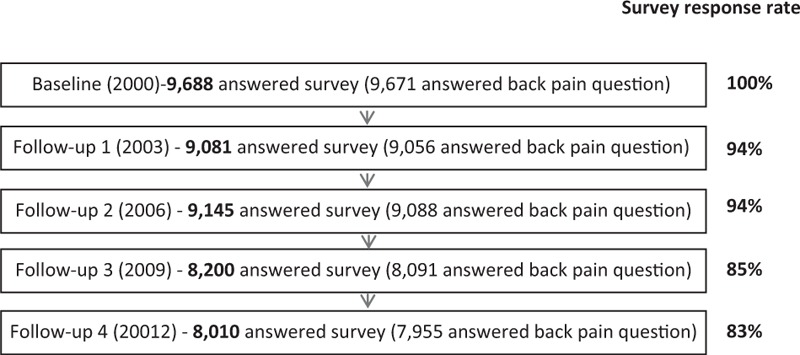
Numbers of women who answered the back pain question at each survey from 2000 to 2012.

The Australian Longitudinal Study of Women's Health (ALSWH) first collected mailed survey data from 3 age cohorts of Australian women in 1996 (young women 1973–1978; middle-aged women 1946–1951; older women 1921–1926). Participants were randomly selected from the national health insurance scheme (Medicare) database (which includes most permanent residents of Australia) with intentional oversampling from rural and remote areas.^23,24^ The women in each cohort have completed surveys at 3-year intervals since 2000. The surveys included questions about a diverse range of issues including health behaviors, health service use, and physical and mental health, as well as social and demographic factors. The young cohort, born between 1973 and 1978, was surveyed in 1996, 2000, 2003, 2006, 2009, and 2012. The present study analyzed data collected from the second survey in year 2000 (because important variables were available in this survey that were not included in the 1996 survey) through to the sixth survey in 2012. Figure 1 shows the numbers and proportions of women who answered a specific question on back pain. Further details of the methods used and sample characteristics have been reported elsewhere^23,24^ and are available on the ALSWH website (www.alswh.org.au). Women who were identified as being currently pregnant at the time of any of the 5 surveys (n = 3010) were excluded from this longitudinal analysis. The Human Research Ethics Committees of the University of Newcastle and the University of Queensland approved the study methods. Written informed consent was obtained from all participants.

### Back Pain

At each survey, women were asked “In the last 12 months have you had back pain?” They were asked to circle one response, which related to the frequency of their back pain: “never,” “rarely,” “sometimes,” or “often.” They were also asked “Did you seek help for this problem?” Participants were asked to circle either “Yes” or “No.” Those who responded “never” or “rarely” having back pain were categorized as “no back pain,” whereas those who responded “sometimes” or “often” were categorized as “back pain.”

### Obesity

Body mass index (BMI) was calculated at each survey from self-reported height and weight and classified as underweight or healthy weight (BMI <25 kg/m^2^), overweight (BMI 25.0–<30 kg/m^2^), or obese (BMI ≥30 kg/m^2^) using the World Health Organization (WHO) criteria. Self-reported weight and height have been shown to be reasonably accurate for the assessment of BMI in a middle-aged female population.^25^

### Physical Activity

Physical activity was assessed at all surveys using questions developed for the national surveillance of physical activity in Australia.^26^ Metabolic equivalent (MET) is a term used to characterize the level of exercise intensity, wherein a single MET is equivalent to the energy utilized by the body at rest. Frequency and duration of brisk walking, and moderate-intensity and vigorous activity were used to calculate total physical activity in MET·minutes per week. Physical activity was categorized based on total MET·minutes per week (none [<40]; low [40–<600]; moderate [600–<1200]; or high [≥1200)].^26^ Inadequate physical activity was determined to be <600 MET minutes per week, the equivalent of 150 minutes per week of moderate-intensity physical activity, as per the Australian and US guidelines for women.^27–29^

### Demographics

Low education status was classified at each survey as having either “no formal qualifications” or “School Certificate (year 10 or equivalent).” Employment status was defined in response to the question “In the last week, how much time in total did you spend doing the following things?” Women were classified as either being “in paid work” (ie, full time, part time, or casual paid work), or “not in paid work” (ie, studying, work without pay, unpaid voluntary work, unable to work, home duties, active leisure, passive leisure). Those in paid work were categorized as “working one to 34 hours per week in paid work” or “working 35 hours or greater per week in paid work.” The presence or absence of depression was identified by the question “Have you ever been told by a doctor that you have depression (not postnatal)?”

### Statistical Analysis

*χ*^2^ tests were used to compare categorical variables in women with and without back pain. Independent samples *t* tests were used to examine the differences in continuous variables across 2 groups. In multivariate analyses, we included variables if the relationship between the variable and the outcome was trending toward significance (*P* ≤ 0.1), or the variable was clinically significant. Since both education and workforce status were highly correlated with each other, we adjusted for education, not workforce status, in all multivariate analyses. Generalized estimating equations (GEEs) were used with logit link and exchangeable correlation structure to evaluate the associations between risk factors such as weight, depression, education, age, and physical activity at baseline with the risk of back pain during the 12-year follow-up period. Time lag analyses were also conducted using repeated measurements of the above risk factors at each survey and back pain reported 1 survey (approximately 3 years) later.^30,31^ All analyses, including interaction testing, were performed using 2-sided tests and a significance level of ≤5% was considered statistically significant. All analyses were performed using Stata SE version 13.0 (StataCorp, College Station, TX).

## RESULTS

**FIGURE 2:**
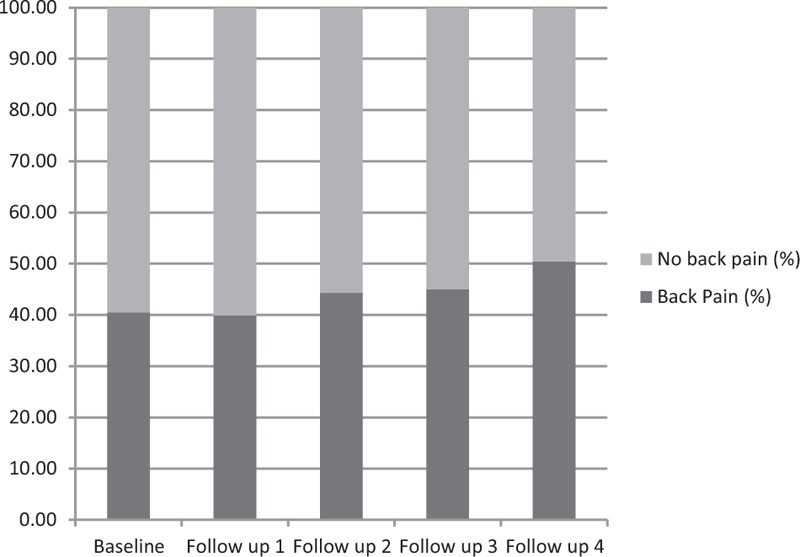
Proportion of women with back pain at each survey (excluding those women currently pregnant at the time of each survey).

In 2000, 9688 women took part in the study. Their median age was 24.6 years (range 20.6–28.6 years). After 12 years, 83% of these women completed the 2012 survey (Figure 1). There were no differences in age, BMI, or back pain prevalence between those who completed only the baseline survey and those who completed all follow-up surveys (n = 5220) (24.6 vs 24.6 years, 23.9 vs 23.7 kg/m^2^, 40.7 vs 39.8%, respectively). The proportions who reported depression (11.6 vs 11.2%), low education status (11.3 vs 7.9%), and participation in inadequate levels of physical activity (76.2 vs 76.6%) were also similar in these 2 groups. The proportions of women with back pain at each of the 5 surveys (excluding those currently pregnant at the time of each survey) are shown in Figure 2. Back pain was common; 41% of the cohort reported having back pain “sometimes” or “often” at baseline, and this increased to 50% by the final survey (Figure 2). Median weight gain across the 12-year study period was 6 kg (interquartile range 11).

Baseline characteristics of the sample (excluding women who were pregnant at that time) are shown in Table 1. Those with back pain tended to be somewhat older, heavier, were more likely to have been diagnosed with depression, more likely to be unemployed, and doing inadequate levels of physical activity than those without back pain. Almost 40% of women with back pain reported seeking help for the condition at the baseline survey.

**TABLE 1:**
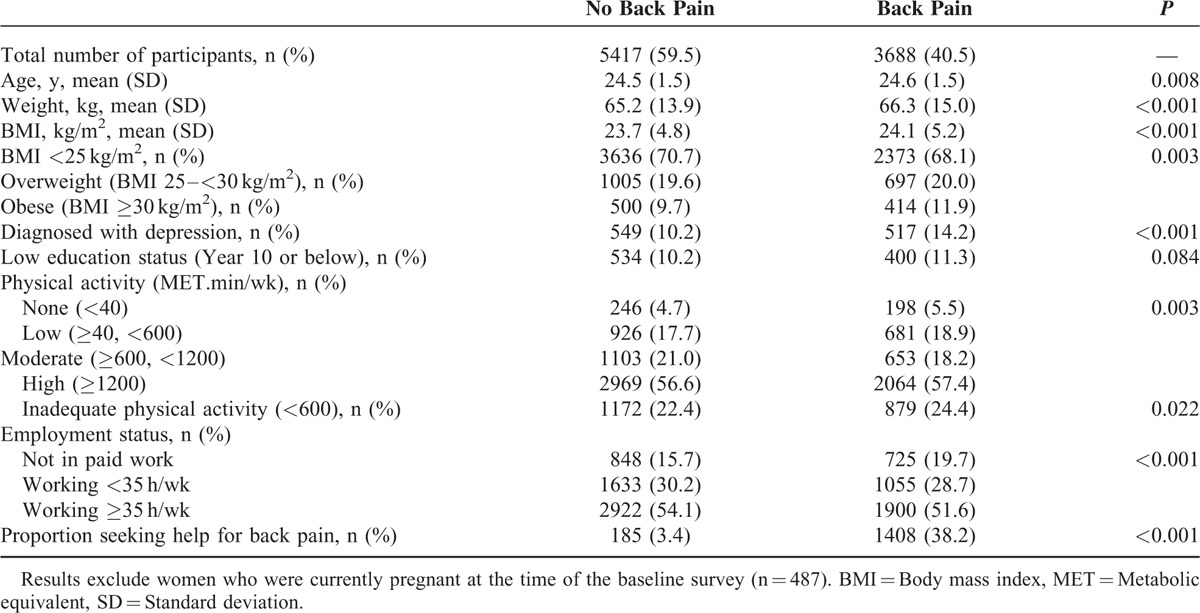
Characteristics of the Study Population at Baseline (n = 9105)

Predictors of future back pain risk are presented in Table 2. Data from women who were pregnant at the time of any of the 5 surveys were excluded from these analyses. In univariate models, baseline age, weight, inadequate physical activity, and depression were associated with back pain over the following 12 years. In multivariate models, for every 5-kg higher weight at baseline, there was a 5% increased risk of back pain during the following 12 years, after adjustment for age, height, education status, physical activity, and depression. Inadequate level of physical activity and diagnosis with depression at baseline were independently associated with a 14% and 17% increased risk of back pain over the following decade, respectively, after adjustment for the remaining variables shown in Table 2 (*P* < 0.001).

**TABLE 2:**
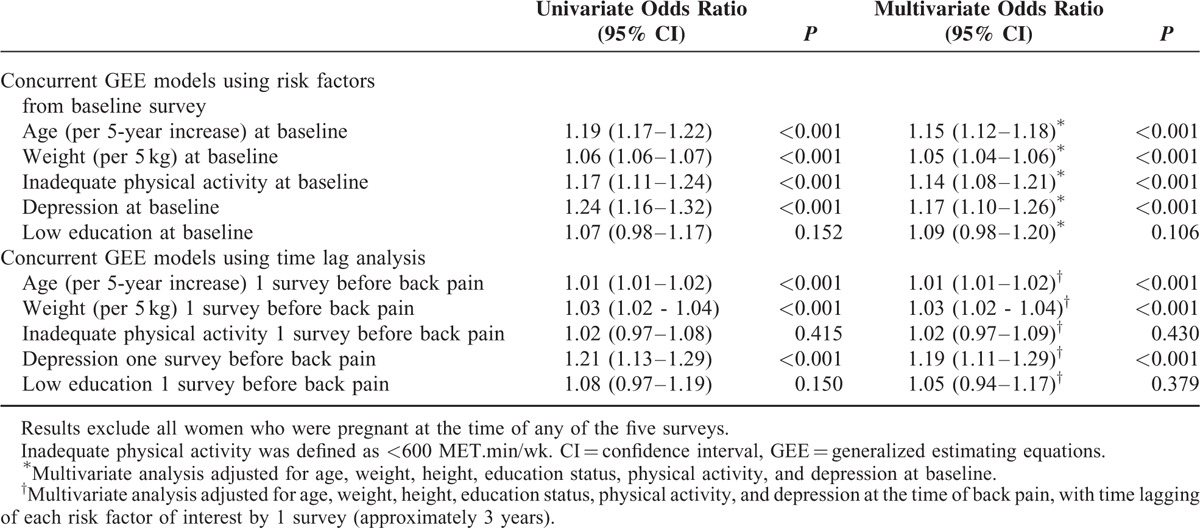
Risk Factors for Back Pain Over 12 Years (Concurrent GEE Models)

When time lag analyses were performed (Table 2), for every 5-kg higher weight, there was a 3% increased risk of back pain 3 years later, after adjusting for age, height, education status, physical activity, and depression at the time of back pain (OR 1.03, 95% confidence interval [CI] 1.02–1.04, *P* < 0.001). A diagnosis of depression was associated with a 19% increased risk of back pain 3 years later, after adjustment for age, weight, height, physical activity, and education status (*P* < 0.001). The effect of inadequate physical activity and low education on back pain Risk 3 years later did not reach statistical significance. Higher age was associated with an increased risk of back pain 3 years later and over the following decade (*P* < 0.001).

Relationships between weight and back pain in the 3-year time lagged models, stratified by physical activity level at the time of reporting back pain, are shown in Table 3. There were no differences in the weight–back pain relationship for different levels of physical activity. Tests for interaction between weight and physical activity on the risk of back pain did not reach statistical significance.

**TABLE 3:**
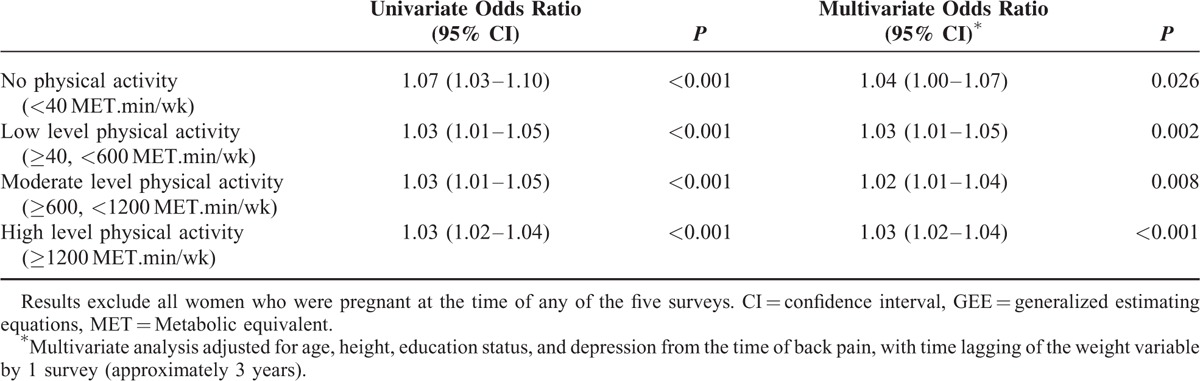
The Association Between Weight and Back Pain, Stratified By Physical Activity Level (GEE Time Lagged Models)—Odds Ratios for Back Pain for Each 5-kg Higher Weight at the Preceding Survey (3 Years Earlier)

When the sample was stratified by BMI (<25 kg/m^2^ and ≥25 kg/m^2^), the effects of weight on back pain were observed in those with BMI ≥25 kg/m^2^, with a trend in those with BMI <25 kg/m^2^. For those with BMI ≥25 kg/m^2^, for every 5-kg higher weight, there was a 4% increase in the risk of back pain, after adjusting for age, height, education, physical activity, and depression (OR 1.04, 95% CI 1.02–1.05, *P* < 0.001). For those with a BMI of <25 kg/m^2^, for every 5-kg higher weight, there was a trend toward a 3% increase in the risk of back pain over the following decade, after adjusting for the above confounders (OR 1.03, 95% CI 1.00–1.06, *P* = 0.072).

## DISCUSSION

In this large community-based cohort of young adult women studied from their mid-20s, to mid-30s, self-reported back pain was common, with 41% reporting back pain at baseline and 50% 12 years later. Self-reported back pain was associated with greater help-seeking behavior and reduced participation in paid work. Higher weight, age, inadequate levels of physical activity, and depression at baseline were all independent predictors of future back pain over the following decade. Higher weight in young women also predicted the risk of back pain 3 years later, with the deleterious effects of weight being similar regardless of physical activity level. This suggests that young women may not be able to mitigate weight-related back pain by undertaking physical activity, without addressing weight.

The 12-month prevalence of self-reported back pain was 41% in this young Australian female population. This is generally consistent with other population-based studies from developed countries,^32–34^ although few studies have specifically examined young adult women. In a study of 2667 adults from 136 general practitioner registers in Britain (including 378 women aged between 20 and 29 years), Walsh et al^32^ reported the prevalence of back pain as 27%, whereas another population-based UK study found that the 12-month prevalence of back pain for women aged between 25 and 34 years was 35%.^33^ Similarly, in a large population-based Danish twin study, approximately 40% of women in their mid-20s had back pain in the past year,^34^ and in a Finnish study of 2575 adults aged 24 to 39 years, 39.5% of women reported back pain in the past 12 months.^35^ Taken together, these data support the notion that back pain is common in young adult women in Europe, and our data confirm similar high rates of back pain in young Australian women.

In this study, we found that higher weight independently predicted the risk of back pain over the next decade. To our knowledge, few longitudinal studies have examined this risk factor in community-based young women. Previous data have largely been from cross-sectional studies^18,19^ or from working populations.^36–38^ Also, past studies are limited by lower rates of obesity and weight gain than are now commonly seen in modern developed countries. In a prospective study of the 1958 British birth cohort, self-reported obesity in women at age 23 was associated with increased incidence of back pain 10 years later.^14^ However, in a subsequent article focussing just on those with incident back pain from 32 to 33 years of age, obesity was no longer found to be a risk factor.^15^ Previous population-based cohort studies, one of 1224 young adults without back pain^17^ and the other of 6554 young adolescent twins,^16^ showed no association between overweight or obesity and nonspecific back pain several years later. This may be in part because of the power of these studies. Although the sample size in the twin study was large, they were relatively young, and being twins, intrapair similarities may further reduce the power to show an effect. However, only 10% of the twin cohort were overweight (classified as BMI >24 kg/m^2^). In contrast, in some large prospective studies of middle-aged adults, obesity in women has been associated with both incident^39^ and chronic^20^ back pain. Therefore, although obesity itself has been shown to be a potential risk factor for back pain in older adults, the data on young women are limited and inconsistent. This ongoing community-based study of a large sample of Australian women provides unique and robust evidence that weight independently predicts future back pain in young women over a 3- and 12-year period, particularly in those with a BMI ≥25 kg/m^2^.

Our study also found that inadequate levels of physical activity (defined as less than 600 MET minutes per week) in young adulthood predicted back pain risk over the following decade. However, inadequate physical activity levels did not appear to predict back pain risk 3 years later. To date, prospective studies that have examined the association between physical inactivity and back pain have yielded inconsistent results.^21,22^ Furthermore, none has focused on a young female population-based sample, nor adequately addressed the interaction between physical activity and weight in a longitudinal study. In a prospective study of young Finnish adults aged 24 to 39 years without back pain at baseline, low levels of physical activity were only associated with incident radiating back pain 6 years later in the obese, but not overweight, adults.^17^ However, in a large study of middle-aged Norwegian adults (mean age >40 years), women who did 1 to 1.9 exercise sessions per week had a reduced risk of chronic back pain at 11-year follow-up, than those who did <1 exercise session per week.^20^ In keeping with these inconsistencies, 2 systematic reviews have concluded that there is conflicting evidence to support an association between physical activity level and back pain.^21,22^ In contrast, our study has demonstrated that inadequate physical activity in young women is an independent predictor of back pain over the following decade. When we examined the relationship between inadequate physical activity and back pain risk 3 years later, it was not statistically significant, suggesting that consistently low levels of physical activity over a longer period may impact back pain risk. Importantly, these longitudinal observational data also suggest that being physically active may not mitigate subsequent weight-related back pain. Further intervention studies are required to explore the role of physical activity in the prevention of back pain at this life stage.

Depression and higher age were found to be independent predictors of back pain 3 years later and over the following decade. Age is a well-known risk factor for back pain.^40^ Even in this young population there is an increased risk of back pain evident with higher age. Depression and psychological distress have, in some prospective studies, been shown to be predictors of back pain,^15,41,42^ but data have been inconsistent and have not focused on young women. For example, a prospective cohort study of 2556 workers aged 35 to 59 years, found that women who felt depressed were not more likely to develop back pain over a 6-year period.^43^ In contrast, Power et al^15^ found that incident back pain in 32- to 33-year-old British women was more likely among those with psychological distress at 23 years of age. Although previously published data have been conflicting in other populations, we found that in this Australian cohort, depression did predict back pain over the following 12 years.

The underlying mechanism of how higher weight, physical inactivity, and depression can contribute to the development of back pain is complex. Both physical inactivity and obesity (specifically fat mass) are independently associated with reduced lumbar intervertebral disc height and high intensity or disability back pain.^44–46^ Moreover, low levels of physical activity have also been associated with increased fat content of paraspinal muscles.^44^ Fat or adipose tissue is metabolically active, releasing a variety of inflammatory mediators, which have been shown to be associated with structural abnormalities in spinal joints,^47^ pain pathways,^48^ and chronic back pain.^49^ There is also evidence suggesting that pain and depression both share common pathophysiologic pathways, including the activation of common neurocircuitries, common neurochemicals such as cytokines, and are associated with common clinical features.^50^ Thus, it is biologically plausible that higher weight, inadequate physical inactivity levels, and depression are independent predictors for back pain, though further research is needed to confirm causation.

This study has some limitations. Obesity was based on self-reported data, which have the potential to introduce bias in terms of under-reporting weight, especially by overweight and obese women.^51^ This is unlikely to explain our positive association between weight and back pain as it is more likely to have biased our results toward the null for the association between weight and back pain, particularly in the overweight and obese women. Although back pain in this study was also self-reported, the question we used identified women with back pain that was sufficient to result in a high proportion of help-seeking and was associated with less likelihood of being in paid work. Although physical activity levels were also self-reported, the survey questions used have been found to have satisfactory validity when compared with accelerometry.^26^ Although depression status was self-reported, similar questions have been found to be reasonably accurate when compared with psychiatric or psychological assessment.^52^ Although loss to follow-up was relatively small in this study, it is possible that the sample has become less representative over time. However, there were no important differences in general characteristics and back pain status between those who completed follow-up and those who did not. Major strengths of this study include the large, community-based population with high participation rates over 12 years, as well as the use of time-lag analysis to demonstrate the temporal relationship between risk factors and back pain. Likewise, a comparison of women who participated in the baseline ALSWH survey (1996) with women in the same age range from the Australian 1996 census has shown that the ALSWH participants were reasonably representative of the general population.^23^

In summary, back pain was common in this community-based, young female cohort, and associated with greater help-seeking behavior and reduced workforce participation. Higher weight, age, inadequate levels of physical activity, and depression were all found to be independent predictors for back pain in young women over the following decade. We also found that the risk of weight-related back pain was not mitigated by increasing physical activity levels. Even though the magnitude of the increased risk in this study was small, the significant burden of back pain on a population level means that these risk factors could potentially have a large impact. Given that over half of our cohort gained 5 kg during the study period, we suggest that a strong emphasis on weight management will be important as a future prevention strategy. Similarly, targeting physical inactivity and improving mental health may be important for the prevention of back pain in young adult women, with intervention studies now needed.
